# The effect of a full-time infection control nursing service in the prevention of multidrug-resistant organism in the orthopedic ward

**DOI:** 10.1186/s12879-022-07331-4

**Published:** 2022-04-07

**Authors:** Yun Yang, Ting-ting Tang, Ji Lin, Chun-lan Gan, Wen-zhi Huang, Yue Fang

**Affiliations:** 1 Department of Orthopaedics, West China Hospital, Sichuan University, Chengdu, Sichuan People’s Republic of China; 2 School of Nursing, West China Hospital, Sichuan University, Chengdu, Sichuan People’s Republic of China; 3 Department of Infection Control, West China Hospital, Sichuan University, Chengdu, Sichuan People’s Republic of China

**Keywords:** Orthopedic ward, Full-time infection control nursing service, Multidrug-resistant organism, Interrupted time-series

## Abstract

**Background:**

Our aim was to evaluate the effect of setting up a full-time infection control nursing service on reducing the prevalence of multidrug-resistant organism (MDRO) in the orthopedic ward.

**Methods:**

From January 2015 to March 2019, routine prevention and control measures were taken for patients infected/colonized with MDRO in this ward, which was set as the pre-intervention period. The intervention period was from April 2019 to June 2021. The study was designed to evaluate whether the establishment of a full-time infection control nursing service could reduce the positive density of MDRO in the hospital by using an interrupted time-series model of a quasi experimental study.

**Results:**

There were 11,759 patients during pre-intervention period and 8142 patients during intervention period. The total number of MDRO isolated before intervention was 177, of which 145 were obtained in hospital and 32 were brought in from outside hospital. The total number of MDRO isolated after intervention was 47, of which 29 were obtained in hospital and 18 were brought in from outside hospital. Before intervention, the positive density of MDRO in the orthopedic ward showed an increasing trend (β_1_ = 0.02, P = 0.003). After intervention, the positive density of MDRO showed a downward trend (β_3_ = − 0.05, P = 0.018).

**Conclusions:**

The establishment of the full-time infection control nursing service in the orthopedic ward can effectively reduce the nosocomial prevalence of MDRO.

## Introduction

In recent years, the prevalence of hospital infection caused by MDRO has become more and more serious. It usually leads to prolonged hospital stay, increased mortality and other serious consequences [1–4], and has been listed as a serious threat by the World Health Organization [5, 6]. Although nosocomial infections account for 4% of all hospital admissions [7], 9% to 20% of critically ill patients develop infections while in the intensive care unit (ICU) [7, 8]. A multicenter study of 2544 patients with prosthetic joint infections found that 24% of them had MDRO infection during early after surgery [9]. In a European multicenter cohort study, 95 of 4131 patients with major trauma had a positive screening for MDRO and the positive MDRO infection correlated with worse outcome [10].

The harsh reality is that few new antibiotics have been developed in the last 30 years [11]. In the context of increasing antimicrobial resistance, the increased demand for intensive care resources and the high risk of nosocomial infection make infection prevention a priority for hospital management. More and more studies have found that infection with multidrug-resistant bacteria can prolong hospitalization time and increase mortality [12–14]. Fortunately, the relevant professional organizations have formulated guidelines to prevent the spread of multidrug-resistant pathogens [15, 16].

In order to reduce nosocomial transmission, many measures, including meticulous hand hygiene, surveillance, contact prevention, patient isolation and environmental disinfection, have been widely adopted and have been recommended in various guidelines and codes [17, 18]. At present, many hospitals have set up a full-time infection control nursing service, which plays an important role in the implementation of infection control measures in clinical departments.

In terms of the actual situation of our hospital, in the preliminary investigation, we found that the prevalence of MDRO in the orthopedic ward was much higher than the average level of the whole hospital. For example, the positive density of hospital-acquired MDRO in trauma orthopedic ward in 2015 was 0.934 cases/per 1000 patient-days (0.658 cases/per 1000 patient-days for the whole hospital). The positive density of hospital-acquired MDRO in trauma orthopedic ward in 2016 was 2.123 cases/per 1000 patient-days, which was far higher than the average level of the whole hospital (0.663 cases/per 1000 patient-days). In addition, as far as we know, there are few studies on the role of the full-time infection control nursing service in the management of MDRO in hospitals. Based on the above considerations, the purpose of this study was to explore the effect of setting up a full-time infection control nursing service in the orthopaedic ward on the prevention and control of MDRO in hospital.

## Materials and methods

### Subjects

**Fig. 1:**
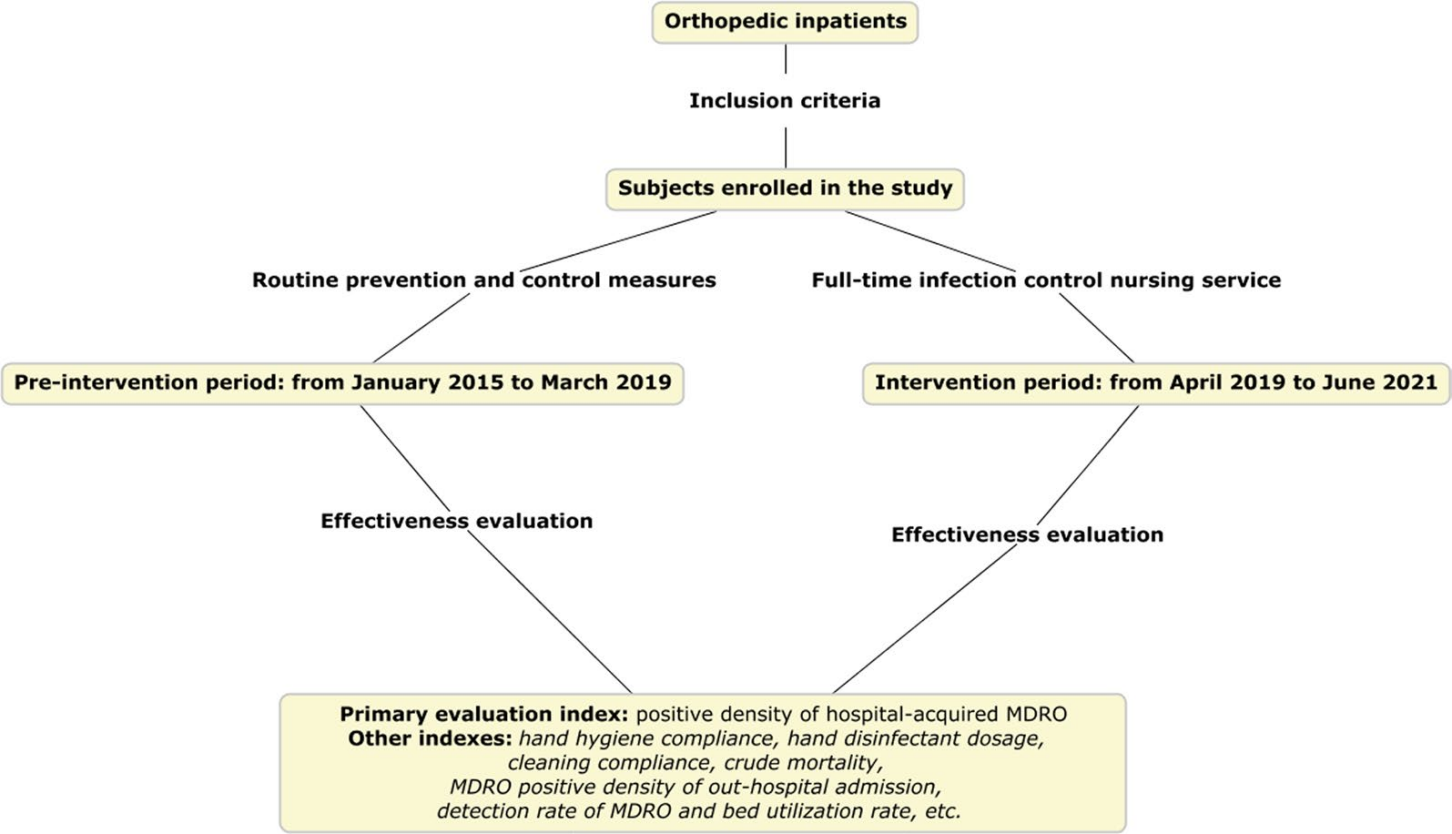
Intervention process and evaluation index of the full-time infection control nursing service. Please refer to relevant literature [19] for the drawing of flow chart

A retrospective analysis was conducted on 19,901 patients who were admitted to the department of orthopaedics at a tertiary center from January 2015 to June 2021. As a university teaching hospital, our hospital had 4300 beds. Among them, there were 68 beds in the trauma orthopedic ward (including 6 ICU beds). These patients were mainly composed of fractures, joint dislocation, vascular, nerve and tendon injuries and multiple injuries, of which nearly half of the patients were open injuries. For critically ill patients, such as those in shock or with severe multiple injuries, damage control theory should be followed. These patients required temporary transition in the trauma ICU and were often supported by some expensive equipment, such as ventilators and real-time cardiac monitoring. Inclusion criteria were (1) age of 18 years or older; (2) at least one microbial culture during their stay; (3) informed consent. MDRO detected before or within 48 h of admission is considered as out-of-hospital acquired MDRO. From January 2015 to March 2019, nursing staff in the ward took routine prevention and control measures for patients with MDRO infection/colonization, which was defined as pre-intervention period. The intervention period lasted from April 2019 to June 2021. The study lasted 78 months from January 2015 to June 2021. The data collected at 78 time points were presented in the unit of month. The study design used an interrupted time-series model from a quasi experimental study to evaluate whether the establishment of a full-time infection control nursing service can reduce the positive density of MDRO (Fig. 1).

### Interventions

In April 2019, a full-time infection control nursing service was established. The post requirements of the service was: (1) regular staff of our hospital with working years ≥ 5 years; (2) bachelor degree or above; (3) supervisor nurse or above title; (4) correct working attitude, careful and serious; (5) strong clinical work and decision-making ability.

The main responsibilities of a full-time infection control nursing service in the prevention and control of multidrug-resistant bacteria in hospitals were as follows: (1) Hand hygiene monitoring: daily monitoring of hand hygiene by doctors, nurses, cleaners, professional attendants and workers in contact with MDRO infected/implanted patients; (2) Environmental disinfection supervision: fluorescent monitoring of the cleanliness of the ward every day, including medical instruments and equipment, bed rails, bed rockers, bedside tables, equipment straps, toilet door handles and faucets, etc.; (3) Monitoring of dressing change process: daily review of dressing change for patients with MDRO infection/colonization, with special attention to the implementation of aseptic techniques during dressing change; (4) Monitoring of exposure and isolation measures, such as timely detection and registration of MDRO positive cases; isolation of patients with MDRO infection/colonization in single room; use of gloves, isolation clothing and other personal protective equipment; Medical supplies such as thermometers, sphygmomanometers and stethoscopes for special use; the limited number of ward rounds; (5) Feedback: if any problem was found during the inspection, immediate face-to-face feedback will be carried out and the morning shift would be reported; (6) Education and training: monthly training on nosocomial infection knowledge for patients and their families; new entrants (including doctors, nurses, training trainees, students) would be trained once upon entering the department, and trained again after 1 month, and assessed when leaving the department. Training staff (including doctors, nurses, workers, cleaners, professional attendants) on nosocomial infection once a quarter.

There were currently six qualified full-time infection control nurses in our unit. On holidays or evenings, they made the handover in advance and arranged one or two nurses to work.

### Case definition

The multidrug-resistant status of the cultured bacteria was defined according to the provisional standard definition of MDRO published in Clinical Microbiology and Infection in 2012. MDRO can be defined as an organism that is resistant to three or more antimicrobials simultaneously [20]. MDRO colonization is defined as the presence of MDRO culture from a microbial specimen without evidence of tissue invasion or inflammation of the body site. MDRO infection is defined as MDRO invading tissue and causing disease [21].

The MDRO in this study included carbapenems resistant *Acinetobacter baumannii* (CRAB), carbapenems resistant *Pseudomonas aeruginosa* (CRPA), carbapenem resistant *Enterobacterales* (CRE), vancomycin resistant *Enterococcus* (VRE) and methicillin resistant *Staphylococcus aureus* (MRSA).

Hospital-acquired MDRO: if the date of test submission exceeded the date of admission by 2 calendar days, it was considered as hospital-acquired MDRO, including hospital-acquired infection and colonization. If the same inpatient developed the same MDRO after multiple tests, only the results of the first test would be retained.

Positive density of hospital-acquired MDRO (cases/per 1000 patient-days) = Total number of positive cases of five types of hospital-acquired MDRO/days actually occupied by beds during the observation period*1000.

Positive rate of MDRO in hospital (%) = Total number of positive cases of five types of hospital-acquired MDRO/number of inpatients during the observation period*100%.

### Data collection

The indicators of hand hygiene compliance, hand disinfectant dosage (about 3.0 ml each time) [22] and cleaning compliance before and after intervention were collected to reflect the implementation of measures. Fluorescent marker was used to mark the surfaces of medical instruments and objects cleaned by nursing staff and cleaning staff, and the assessment of cleaning compliance was performed through observing the residual fluorescence. The inpatients' crude mortality, MDRO positive density of out-hospital admission, detection rate of MDRO and bed utilization rate before and after intervention were collected to reflect the comparability before and after intervention. The positive density of hospital-acquired MDRO was taken as the primary evaluation index. We calculated each month for the positive density of hospital-acquired MDRO. The numerator of this index was the total number of positive cases of five types of hospital-acquired MDRO. If the same kind of MDRO was produced in a patient after multiple test, only the results of the first test would be retained (that is, the number of new cases of MDRO in each patient would be counted only at one time point and not at other time points). The denominator of this index was days actually occupied by beds, which were the total number of hospitalized days of all patients in each month, obtained from department report data.

### Statistical analysis

SPSS25.0 software (SPSS Chicago, IL, USA) was used for statistical analyses. In descriptive analysis, qualitative data were expressed as frequency (rate), and quantitative data were expressed as median (25th–75th percentile). For qualitative data, the Chi-square test or Fisher's exact test was used. For quantitative data, skewness distribution was observed after normality test, and Mann–Whitney U test was used. Poisson's test was used to compare the two true rates. The interrupted time-series model was used to analyze whether the change in the positive density of hospital-acquired MDRO at the intervention point level and the slope of the change over time after intervention were statistically significant compared with that before intervention. The fitted piecewise multiple linear regression model was shown as follows:

$$Y_{t} = \beta_{0} + \beta_{1} X_{1} + \beta_{2} X_{2} + \beta_{3} X_{3} + \varepsilon_{t}$$

In this formula, X_1_ was the counting time variable, X_1_ = 1, 2, 3…, 78 (78 points in total from January 2015 to June 2021); X_2_ stood for intervention, X_2_ = 0 before intervention, X_2_ = 1 after intervention; X_3_ represented the slope, which represented the slope before intervention, and was the level change and slope change, which were used to test whether the three regression coefficients were statistically significant. At the same time, Durbin–Watson method was used to test whether the time series had autocorrelation. If there was autocorrelation, generalized least square estimation was used. The test level was α = 0.05. The sensitivity analysis was carried out with the positive rate of hospital-acquired MDRO as the outcome index. In addition, the monthly data of four indicators including the crude mortality of inpatients, MDRO positive density of out-hospital admission, bed utilization rate and hand disinfectant dosage were incorporated into the interrupted time-series model as covariates to further explore the robustness of the model.

## Results

### Composition of hospital-acquired MDRO specimen types

There were 11,759 patients during pre-intervention period and 8142 patients during intervention period. A total of 10,512 and 4459 samples were collected during pre-intervention period and the intervention period respectively. Specimen types of hospital-acquired MDRO in the orthopaedic ward included secretions, ascites, drainage fluid, sputum and others. Among the positive cases of hospital-acquired MDRO, there were 145 cases in the pre-intervention period, and 104 cases were male (71.72%). Of the 29 cases in the intervention period, 23 cases were male (79.31%). Chi-square test showed no statistically significant difference (Chi-square value 0.705, P = 0.401). Among hospital-acquired MDRO in orthopaedic ward, CRAB accounted for 45.40% (79/174). MRSA followed, accounting for 21.26 (37/174). Among different specimen types, the proportion of secretions/ascites/drainage fluid was the highest, up to 74.71% (130/174) (Table 1).

**Table 1:** Specimen types of hospital-acquired MDRO in the orthopaedic ward

MDRO	Pre-intervention period (n)				Intervention period (n)			
	Secretions/ascites/drainage fluid	Phlegm	Others	Total	Secretions/ascites/drainage fluid	Phlegm	Others	Total
CRAB	50	14	3	67	8	4	0	12
MRSA	24	5	0	29	7	0	1	8
CRPA	22	4	2	28	0	0	0	0
CRE	14	2	5	21	5	1	3	9
VRE	0	0	0	0	0	0	0	0
Total	110	25	10	145	20	5	4	29

### Baseline comparability between pre-intervention and intervention periods

There were no significant differences in the detection rate of MDRO, the positive density of out-hospital admission and the mortality of inpatients in the orthopedic ward between pre-intervention period and intervention period (P > 0.05). It could be considered that these variables were comparable in the two periods. The bed utilization rate decreased from 112.79% in the pre-intervention period to 90.20% in the intervention period, indicating that the situation of additional beds in the ward was improved. Since the rate exceeded 100% in the pre-intervention period, no statistical test was conducted (Table 2).

**Table 2:** Comparison of characteristics between pre-intervention period and intervention period

Characteristics	Pre-intervention period	Intervention period	χ^2^/u	P
Detection rate of MDRO (%)				
CRAB	62.22% (56/90)	67.74% (21/31)	0.30	0.582
MRSA	29.07% (25/86)	40.54% (15/37)	1.55	0.213
CRPA	20.97% (13/62)	8% (2/25)	1.29	0.256
CRE *Klebsiella pneumoniae*	26.67% (16/60)	20% (7/35)	0.54	0.464
CRE *Escherichia coli*	1.05% (1/95)	1.92% (1/52)	/	1.000
CRE *Enterobacter cloacae*	4.94% (4/81)	6.25% (2/32)	/	1.000
VRE *Enterococcus faecalis*	0% (0/32)	0% (0/10)	/	/
VRE *Enterococcus faecium*	0% (0/30)	0% (0/10)	/	/
MDRO positive density of out-hospital admission (cases/per 1000 patient-days)	0.269 (32/118956)	0.357 (18/50416)	− 0.91	0.362
Mortality of inpatients (%)	0.15% (18/11759)	0.16% (13/8142)	0.01	0.908
Bed utilization rate (%)	112.79% (118,956/105468)	90.20% (50,416/55896)	/	/

*Enterococcus faecalis* and *Enterococcus faecium* are the most clinically relevant subspecies of enterococci [23]

### Implementation of interventions

Both hand hygiene compliance and cleaning compliance increased after intervention, and the difference was statistically significant (P < 0.05) (Table 3).

**Table 3:** Comparison of compliance between pre-intervention period and intervention period

Measures	Pre-intervention period	Intervention period	χ^2^/Z	P
Hand hygiene compliance (%)	51.54% (451/875)	74.23% (625/842)	94.39	< 0.001
Hand disinfectant dosage (ml/per bed. per day)^a^	39.13 (35.19–45.97)	48.64 (35.66–55.01)	− 2.23	0.024
Cleaning compliance (%)	71.20% (581/816)	95.96% (783/816)	182.17	< 0.001

^a^Median (25th to 75th percentile)

### Analysis of the effectiveness of interventions

**Fig. 2:**
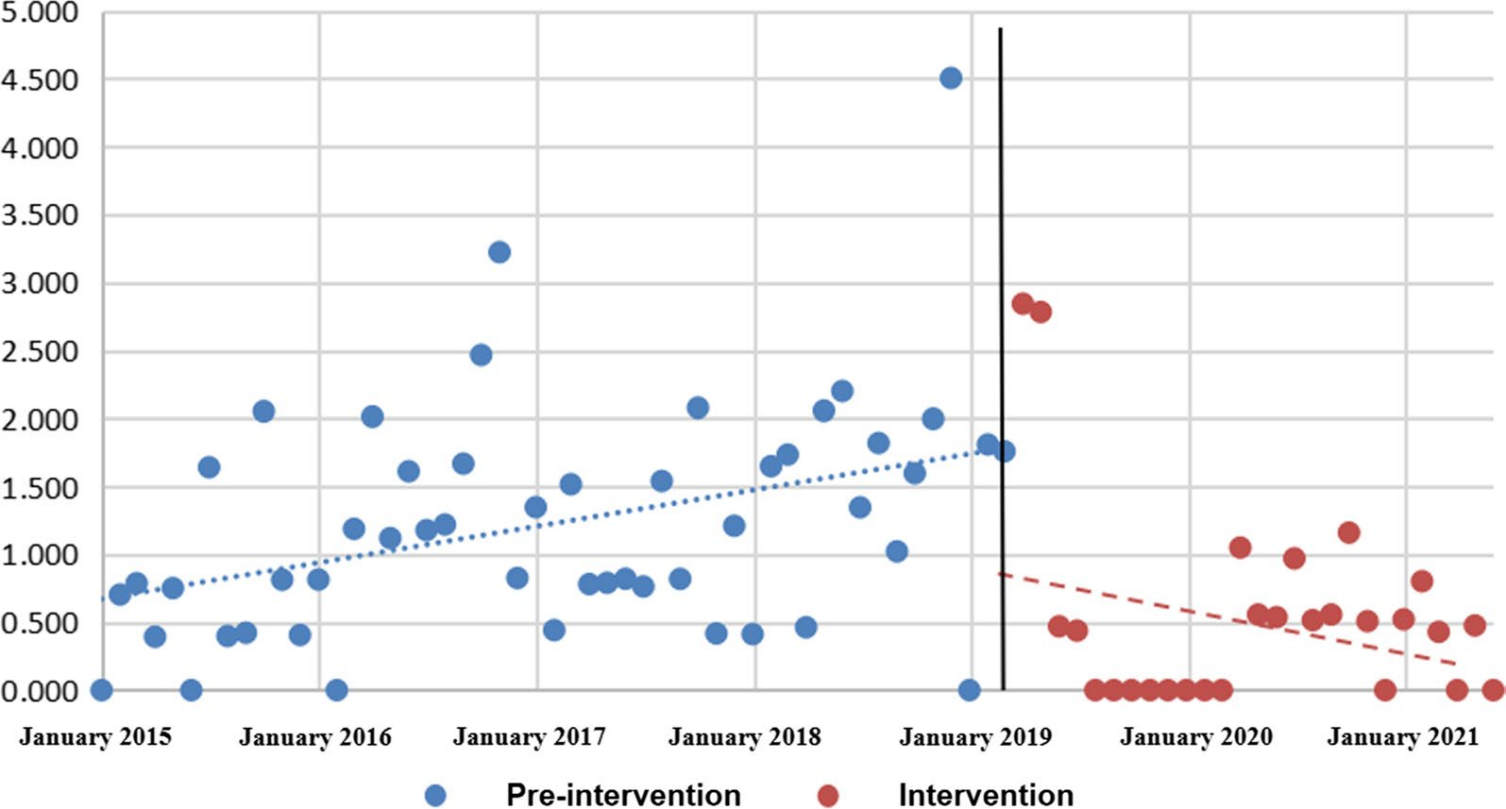
Interrupted time-series model for positive density of hospital-acquired MDRO

With the positive density of hospital-acquired MDRO as the dependent variable and the counting time variable (X_1_), intervention measures (X_2_) and slope (X_3_) as the independent variables, the fractional multiple linear regression model was fitted. The DW value in Durbin–Watson test was 1.85 (close to 2), indicating that there was no first-order autocorrelation in this time series. The specific results were shown in Table 4 and Fig. 2. The slope before intervention was 0.02, and P = 0.003, indicating that the positive density of hospital-acquired MDRO in the orthopedic ward showed an upward trend before intervention. There was no statistical difference (P = 0.195), indicating that there was no statistically significant change in the positive density of hospital-acquired MDRO when the full-time infection control nursing service was set up. The slope change after intervention was − 0.05, so the slope after intervention was 0.02–0.05 = − 0.03. After intervention, the positive density of hospital-acquired MDRO showed a downward trend, and the results were statistically different (P = 0.018).

**Table 4:** Interrupted time-series analysis for positive density of hospital-acquired MDRO

Variable	β	SE	t	P
Constant	0.65	0.22	2.96	0.004
X_1_	0.02	0.01	3.03	0.003
X_2_	1.66	1.27	1.31	0.195
X_3_	− 0.05	0.02	− 2.42	0.018

### Sensitivity analysis

The sensitivity analysis was conducted with the positive rate of hospital-acquired MDRO as the outcome index, and the results were similar. The regression coefficients β_1_ and β_2_ were statistically significant, with P values of 0.009 and 0.016, respectively, indicating that the positive rate of hospital-acquired MDRO in the orthopedic ward showed an upward trend before intervention, and was a downward trend after intervention.

In addition, four covariables were included into the interrupted time-series model to further explore the robustness of the model. The results were similar, and the regression coefficients β_1_ and β_3_ were statistically significant (Table 5).

**Table 5:** Sensitivity analysis after the inclusion of four covariables

Variable	β	SE	t	P
Constant	− 0.48	1.02	− 0.47	0.637
X_1_	0.02	0.01	3.10	0.003
X_2_	1.77	1.39	1.27	0.207
X_3_	− 0.05	0.02	− 2.23	0.029
Crude mortality of inpatients	0.03	0.36	0.07	0.944
MDRO positive density of out-hospital admission	0.04	0.24	0.16	0.871
Bed utilization rate	0.01	0.01	1.14	0.257
Hand disinfectant dosage	0.00	0.01	0.40	0.692

## Discussion

In recent years, with the continuous reform of medical practices, the importance and role of nursing work has become increasingly prominent. Many hospitals are making efforts to strengthen the prevention and control of nosocomial infection, and have implemented many new measures to improve the prevention and control effect of nosocomial infection through effective management. A study has shown that training and adding sensory-controlled nurses can effectively reduce the incidence of nosocomial infection [24]. However, part-time infection control nurses undertake heavy clinical nursing work as well as infection control work [25], which is obviously not conducive to the supervision of infection control and the implementation of corresponding measures. At the same time, the orthopedic ward is prone to nosocomial cross infection due to the large number of open injuries and weakened immunity of inpatients, which will not only affect the rehabilitation process of patients, but also cause a lot of unnecessary waste of medical resources. After a comprehensive evaluation, the orthopedic ward of our hospital set up a full-time infection control nursing service. All their responsibilities and tasks revolved around nosocomial infection prevention and control, with emphasis on promoting the implementation of nosocomial infection measures.

The interrupted time-series model has the highest demonstration strength in the design of quasi experiment, and its data are collected from multiple time points before and after the intervention. Data were collected at 78 time points in this study, including 51 date in the pre-intervention period and 27 date in the post-intervention period, meeting the sample size requirements of a single group of interrupted time-series model (20 observation data before and after intervention) [26]. In this study, compared with the pre-intervention period, there were fewer time points and fewer patients and sample size in the post-intervention. This could be explained in the following aspect. The intervention was initiated in April 2019 as the MDRO was monitored from 2015 to 2019 on a continuous upward trend. However, due to time constraints, the data of the intervention period was less than that of the pre-intervention period, but we had extended the intervention period to June 2021 as far as possible. Domestic studies on the intervention of nosocomial infection and multidrug-resistant bacteria infection/colonisation mostly adopt self-controlled design before and after [27], with relatively low demonstration intensity, while the interrupted time-series model can make full use of the monitoring data of nosocomial infection to obtain reliable results. This study mainly evaluated the range of changes in the positive density of hospital-acquired MDRO at the beginning of the intervention and the long-term trend after the implementation of the intervention. β_2_ reflected the instant effect of intervention on the level change of the positive density of hospital-acquired MDRO in the interrupted time-series. There was no statistically significant change in the positive density of MDRO in the hospital (β_2_ = 1.66, P = 0.195) when the full-time infection control nursing service was just set up. The results indicated that the intervention did not have an immediate effect. In spite of this, the long-term trend showed a downward trend, indicating the effectiveness of the intervention. In addition, in order to reduce the subjectivity of the results, we did not use MDRO infection rate, but hospital acquisition as the outcome indicator, and sensitivity analysis was also performed to ensure the robustness of the results.

The full-time infection control nursing service have strengthened the supervision of various nosocomial infection measures in clinical departments. Among the prevention and control measures implemented in the wards, in addition to the national requirements, some more detailed measures have been formulated by our hospital. For example, when the medical team makes ward rounds, there are no more than three people. Such restrictions cannot be found in international guidelines. But our country has its own national conditions, coupled with our hospital as a teaching hospital, the number of ward rounds often reach more than 10 to 20 people. If these people are crowded in a ward setting and not taken seriously, they can cause the nosocomial transmission of MDRO. For example, when wearing isolation clothes in our hospital is suitable for large-area contact with patients, and the large-area contact is defined (such as turning over, dressing change when the maximum diameter of the wound ≥ 5 cm, etc.), so as to ensure the appropriate and not excessive use of protective equipment.

There are several limitations that should be acknowledged. Firstly, it was difficult to infer causality in the quasi-experimental design, and the changes in level and trend after intervention may also be caused by other potential variables. Secondly, the interventions implemented by the full-time infection control nursing service were clustered and it was difficult to explain the effect of a specific measure.

## Conclusion

We believe that it is of great significance to set up a full-time infection control nursing service in the orthopedic ward, which can effectively reduce the nosocomial prevalence of MDRO. In the future, we will continue to push the service to become more richer and more conducive to improving patient satisfaction, rather than being limited to a simple concept.

## Data Availability

The datasets generated during the current study are not publicly available due to the limitations of hospital regulations but are available from the corresponding author on reasonable request.

## Abbreviations

MDRO: Multidrug-resistant organism
ICU: Intensive Care Unit
CRAB: Carbapenems resistant *Acinetobacter baumannii*
CRPA: Carbapenems resistant *Pseudomonas aeruginosa*
CRE: Carbapenem resistant *Enterobacterales*
VRE: Vancomycin resistant *Enterococcus*
MRSA: Methicillin resistant *Staphylococcus aureus*
